# Activation of TC10-Like Transcription by Lysine Demethylase KDM4B in Colorectal Cancer Cells

**DOI:** 10.3389/fcell.2021.617549

**Published:** 2021-06-23

**Authors:** Baoyu Chen, Yuwen Zhu, Junliang Chen, Yifei Feng, Yong Xu

**Affiliations:** ^1^Key Laboratory of Targeted Intervention of Cardiovascular Disease and Collaborative Innovation Center for Cardiovascular Translational Medicine, Department of Pathophysiology, School of Basic Medical Sciences, Nanjing Medical University, Nanjing, China; ^2^Department of Pathophysiology, Wuxi Medical School, Jiangnan University, Wuxi, China; ^3^Department of Colorectal Surgery, The First Hospital Affiliated With Nanjing Medical University, Nanjing, China; ^4^Department of General Surgery, The First School of Clinical Medicine, Nanjing Medical University, Nanjing, China; ^5^Institute of Biomedical Research, Liaocheng University, Liaocheng, China

**Keywords:** transcriptional regulation, epigenetics, H3K9 methylation, histone demethylation, lysine demethylase

## Abstract

Malignant colorectal cancers (CRCs) are characterized by enhanced migration and invasion thus acquiring the ability to metastasize. We have previously shown that the small GTPase TC10-like (TCL) contributes to aggressive migration and invasion in malignant CRC cells. TCL expression is differentially expressed in CRC cells and can be upregulated by hypoxia although the underlying epigenetic mechanism is not fully appreciated. Here, we report that differential TCL expression in CRC cells appeared to be associated with histone H3K9 methylation. RNAi screening revealed that the lysine demethylase KDM4B was essential for TCL transcription in CRC cells. KDM4B interacted with and was recruited by the sequence-specific transcription factor ETS-related gene 1 (ERG1) to the TCL promoter to activate transcription. Mechanistically, KDM4B mediated H3K9 demethylase facilitated the assembly of pre-initiation complex (PIC) on the TCL promoter. KDM4B knockdown attenuated migration and invasion of CRC cells. Importantly, KDM4B expression was upregulated in human CRC specimens of advanced stages compared to those of lower grades and associated with poor prognosis. Together, these data uncover a novel epigenetic mechanism underlying malignant transformation of CRC cells and suggest that KDM4B may be considered as a therapeutic target in CRC intervention.

## Introduction

Colorectal cancer (CRC) ranks the third in terms of incidence and mortality rate in the United States regardless of gender (Siegel et al., 2020). It is estimated that newly diagnosed CRC cases will increase by approximately 1,500,000 each year causing ~50,000 cancer-related deaths. Malignant CRC cells differentiate from benign CRC cells by their aggressive proliferation, resistance to chemotherapy, and the ability to migrate and adapt to distal sites. The acquisition of these new phenotypes by malignant CRC cells is underscored by the overhaul of cellular transcriptome. Transcriptomic analyses performed with cultured CRC cells and human specimens reveal that distinctive pathways involved in cell–cell adhesion, apoptosis, stress response, and cell cycling are altered during malignant transformation (Wang et al., 2017; Park et al., 2020). These studies have provided biomarkers for CRC diagnosis and yielded valuable insights for personalized CRC treatment (Cho et al., 2019; Kim et al., 2019; Zhou et al., 2020; Zhu et al., 2020).

Transcriptional events in mammalian cells are programmed by the coordinated actions between sequence-specific transcription factors and epigenetic co-factors. The epigenetic machinery, including histone and DNA modifying enzymes (Audia and Campbell, 2016), chromatin remodeling proteins (Kaur et al., 2019; Centore et al., 2020), histone variants (Cavalieri, 2020; Scott and Campos, 2020), and non-coding regulatory RNAs (Fazi and Fatica, 2019; Zhan et al., 2020), plays a key role in the pathogenesis of CRC development and progression (Lao and Grady, 2011). Generally speaking, promoters of actively transcribed genes are marked by high levels of acetylated histones (Eberharter and Becker, 2002) and methylated H3K4 (Shilatifard, 2012). On the contrary, methylated H3K9, methylated H3K27, and methylated H420 are indicative of transcriptional repression (Jambhekar et al., 2019). A long held view used to be that histone methylation was a relatively stable modification and could not be actively erased from the chromatin. The discovery of histone demethylases, while making this notion completely obsolete, represents a giant step forward for the epigenetic field (Shi and Tsukada, 2013). Thus far, more than a dozen different lysine demethylases (KDMs) have been identified and characterized with highly selective substrate specificities. The roles of these KDMs in CRC development and progression are being actively pursued (Huang et al., 2017).

TC10-like (TCL), alternatively termed RHOJ, is a member of the RHO small GTPase superfamily. TCL was initially identified by screening the human and murine cDNA database for expressed sequence tags (ESTs) that share homology with the TC10 RHO GTPase (Vignal et al., 2000). Further characterization by Heath and colleagues suggests that TCL is preferentially expressed in vascular endothelial cells and is involved in tube formation under physiological conditions (Kaur et al., 2011). Of interest, it is proposed that TCL likely contributes to vasculogenesis by promoting the migration of endothelial cells (Leszczynska et al., 2011). Altered TCL expression can be detected in a wide range of cancer cells including melanoma cancer cell (Ho et al., 2012), gastric cancer (Kim et al., 2016a), ovarian cancer (Kaur et al., 2011), and breast cancer (Kim et al., 2014). We have previously shown that TCL expression levels are elevated in highly malignant CRC and predict poor prognosis (Chen et al., 2020a). Building on this discovery, we further investigated the epigenetic mechanism whereby histone (de)methylation contributes to differential TCL expression in CRC cells. Our data as presented here suggest that the lysine demethylase KDM4B interacts with ETS-related gene 1 (ERG1) to activate TCL transcription by facilitating the assembly of RNA Pol II PIC on the TCL promoter.

## Materials and Methods

### Cell Culture

Human CRC cells (HT29, Caco2, SW480, and HCT116) were maintained in RPMI1640 medium supplemented with 10% FBS as previously described (Chen et al., 2020a; Sun et al., 2020). FLAG-tagged ERG1 (Yuan et al., 2011) and Myc-tagged KDM4B have been previously described (Li et al., 2019a). Small interfering RNA (siRNA) sequences were purchased from Dharmacon: for human ERG1, GTGACTGTTTGGCTTATAATT; and for human KDM4B#1, CAAATACGTGGCCTACATATT; for human KDM4B#2: CTCTTCACGCAGTACAATATT. Transient transfections were performed with Lipofectamine 2000 (Invitrogen) as previously described (Wu et al., 2020a; Yang et al., 2020a,b).

### RNA Isolation and Real-Time PCR

RNA was extracted with the RNeasy RNA isolation kit (Qiagen) as previously described (Fan et al., 2020; Li et al., 2020b,c,d). Reverse transcriptase reactions were performed using a SuperScript First-strand Synthesis System (Invitrogen). Real-time PCR reactions were performed on an ABI Prism 7500 system with the following primers: TCL, 5'-CGGCTGCAATGGACATGAG-3' and 5'-GGCACGTATTCCTCTGGGAAG-3'. Ct values of target genes were normalized to the Ct values of a house keekping control gene (18 s, 5'-CGCGGTTCTATTTTGTTGGT-3' and 5'-TCGTCTTCGAAACTCCGACT-3') using the ∆∆Ct method and expressed as relative mRNA expression levels compared to the control group which is arbitrarily set as one. All experiments were performed in triplicate wells and repeated three times.

### Protein Extraction, Immunoprecipitation, and Western Blot

Whole cell lysates were obtained by re-suspending cell pellets in RIPA buffer (50 mM Tris pH7.4, 150 mM NaCl, and 1% Triton X-100) with freshly added protease inhibitor (Roche) as previously described (Chen et al., 2020c; Dong et al., 2020; Lv et al., 2020; Mao et al., 2020a,b; Wu et al., 2020b; Hong et al., 2021). Specific antibodies or pre-immune IgGs (P.I.I.) were added to and incubated with cell lysates overnight before being absorbed by Protein A/G-plus Agarose beads (Santa Cruz). Precipitated immune complex was released by boiling with 1X SDS electrophoresis sample buffer. Western blot analyses were performed with anti-ERG1 (Cell Signaling Tech, 97249), anti-KDM4B (Cell Signaling Tech, 8,639), anti-TCL (Sigma, HPA003050), and anti-β-actin (Sigma, A2228) antibodies. All experiments were repeated three times.

### Chromatin Immunoprecipitation

Chromatin Immunoprecipitation (ChIP) assays were performed essentially as described before (Fan et al., 2019; Kong et al., 2019a,b; Li et al., 2019b,c,d,e; Liu et al., 2019; Lu et al., 2019; Shao et al., 2019; Weng et al., 2019). In brief, chromatin in control and treated cells were cross-linked with 1% formaldehyde. Cells were incubated in lysis buffer (150 mM NaCl, 25 mM Tris pH 7.5, 1% Triton X-100, 0.1% SDS, and 0.5% deoxycholate) supplemented with protease inhibitor tablet and PMSF. DNA was fragmented into ~500 bp pieces using a Branson 250 sonicator. Aliquots of lysates containing 200 μg of protein were used for each immunoprecipitation reaction with anti-acetyl H3 (Millipore, 06-599), anti-trimethyl H3K4 (Millipore, 07-473), anti-dimethyl H3K9 (Millipore, 07-441), anti-RNA Polymerase II (Santa Cruz, sc-899), anti-KDM4B (Cell Signaling Tech, 8,639), anti-trimethyl H3K27 (Millipore, 04-449), anti-trimethyl H4K20 (Abcam, ab9053), anti-TBP (Abcam, ab818), anti-TFIID (Santa Cruz, sc-273), anti-RNA Pol II (Santa Cruz, sc-899), anti-CTD Ser5 (Active Motif, 61085), or pre-immune IgG. For re-ChIP, immune complexes were eluted with the elution buffer (1% SDS, 100 mM NaCO_3_), diluted with the re-ChIP buffer (1% Triton X-100, 2 mM EDTA, 150 mMNaCl, and 20 mMTris pH 8.1), and subject to immunoprecipitation with a second antibody of interest. Precipitated genomic DNA was amplified by real-time PCR with the following primers: TCL proximal promoter, 5'-AGTGGGACCCCTAGTGTTTTC-3' and 5'-AGGAAATCATGGGTTTCCTG-3'; TCL distal promoter, 5'-GGGTTCCTATAAATACGGACTGC-3' and 5'-CTGGCACTGCACAAGAAGA-3'. A total of 10% of the starting material is also included as the input. Data are then normalized to the input and expressed as percent recovery relative the input. All experiments were performed in triplicate wells and repeated three times.

### Scratch-Wound Healing/Migration Assay

Wound healing assay was performed as previously described (Yang et al., 2019b; Zhao et al., 2019). Cells were re-suspended in serum-free media. When the cells reached confluence, scratch wound was created by using a sterile micropipette tip. Cell migration was measured 24 h after the creation of the wound and calculated by Image Pro. Data were expressed as percent migration compared to control arbitrarily set as 100%.

### Boyden Chamber Invasion Assay

Transwell assay was performed as previously described (Yang et al., 2019a). Twenty-four-well inserts (Costar) with 10 μg/ml Matrigel (Sigma) were used for invasion assays. Cells were re-suspended in serum-free media and plated into the upper chamber with the lower chamber filled with complete media. Following exposure to indicated stimuli, the cells on the upper chamber were removed. Invaded cells were stained with 0.1% crystal violet and counted. Data were expressed as percent invasion compared to control arbitrarily set as 100%.

### DNA Affinity Pull-Down and *in vitro* Demethylase Assay

DNA affinity pull-down assay was performed as previously described (Chen et al., 2020b). Nuclear proteins (~100 μg) were incubated with biotin-labeled RhoJ DNA probe at room temperature for 1 h in 1X binding buffer (20 mM HEPESpH7.9, 0.1 mM EDTA, 4% glycerol, 2 mM DTT) supplemented with BSA (50 μg per reaction), poly-dIdC, and sonicated salmon sperm DNA (100 μg per reaction). DNA-protein complexes formed were then captured by incubating with the streptavidin beads (Promega) for 1 h at 4°C on a shaking platform. Ternary complex (biotin-labeled DNA-protein-streptavidin) was washed three times with 1X binding buffer supplemented with 0.01% Triton X and 100 mM KCl for 10 min each wash. Eluates from the DNA affinity pull-down experiments were used for *in vitro* HDM assay using a commercially available kit (EpiQuik, Epigetek) per vendor recommendations. The assay was performed in triplicate wells and repeated three times. Data were normalized to the control group and expressed as relative HDM activity.

### Statistical Analysis

Sample sizes reflected the minimal number needed for statistical significance based on power analysis and prior experience. Two-tailed Student’s *t* text was performed using an SPSS package. Unless otherwise specified, values of *p* smaller than 0.05 were considered statistically significant.

## Results

### Histone H3K9 Methylation Status Determines Differential TCL Expression in CRC Cells

**Figure 1:**
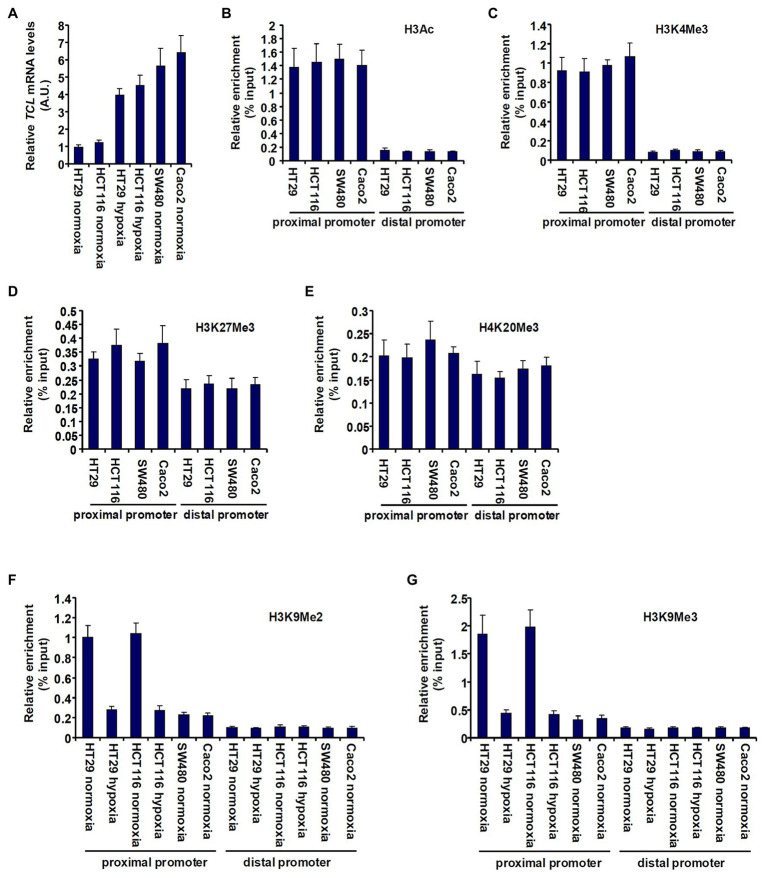
Histone H3K9 methylation status determines differential TC10-like (TCL) expression in CRC cells. **(A)** colorectal cancer (CRC) cells were exposed to hypoxia or normoxia for 24 h. TCL expression levels were examined by qPCR. **(B–E)** Chromatin immunoprecipitation (ChIP) assays were performed with anti-pan-acetyl H3 **(B)**, anti-H3K4Me3 **(C)**, anti-H3K27Me3 **(D)**, and anti-H4K20Me3 **(E)** in CRC cells. **(F,G)** CRC cells were exposed to hypoxia or normoxia for 24 h. ChIP assays were performed with anti-H3K9Me2 **(F)** and anti-H3K9Me3 **(G)**.

First, we sought to determine whether TCL expression in different CRC cells could be accounted for by differential histone modifications surrounding the proximal *TCL* promoter. As shown in Figure 1A, TCL expression levels were lower in HT29 and HCT116 cells than in SW480 cells and Caco2 cells but could be augmented by exposure to hypoxia, consistent with our previous report (Chen et al., 2020a). ChIP assays performed with different anti-modified histone antibodies showed that levels of pan-acetylated H3 (Figure 1B) and trimethylated H3K4 (Figure 1C), two prototypical markers demarcating transcriptionally active chromatin, were comparable across the proximal *TCL* promoter in the four CRC cells. On the other hand, when levels of repressive hisone modifications were examined by ChIP it was discovered that trimethylated H3K27 (Figure 1D) and trimethylated H4K20 (Figure 1E) were similarly enriched surrounding the TCL promoter. On the contrary, dimethylated H3K9 (Figure 1F) and trimethylated H3K9 (Figure 1G) surrounding the proximal, but not the distal, TCL promoter were much higher in HT29 cells and HCT116 cells than in SW480 cells and Caco2 cells. Further, hypoxia treatment downregulated di- and tri-methylated H3K9 levels on the TCL promoter in HT29 cells and HCT116 cells consistent with the changes in TCL expression.

### KDM4B Regulates TCL Transcription in CRC Cells

**Figure 2:**
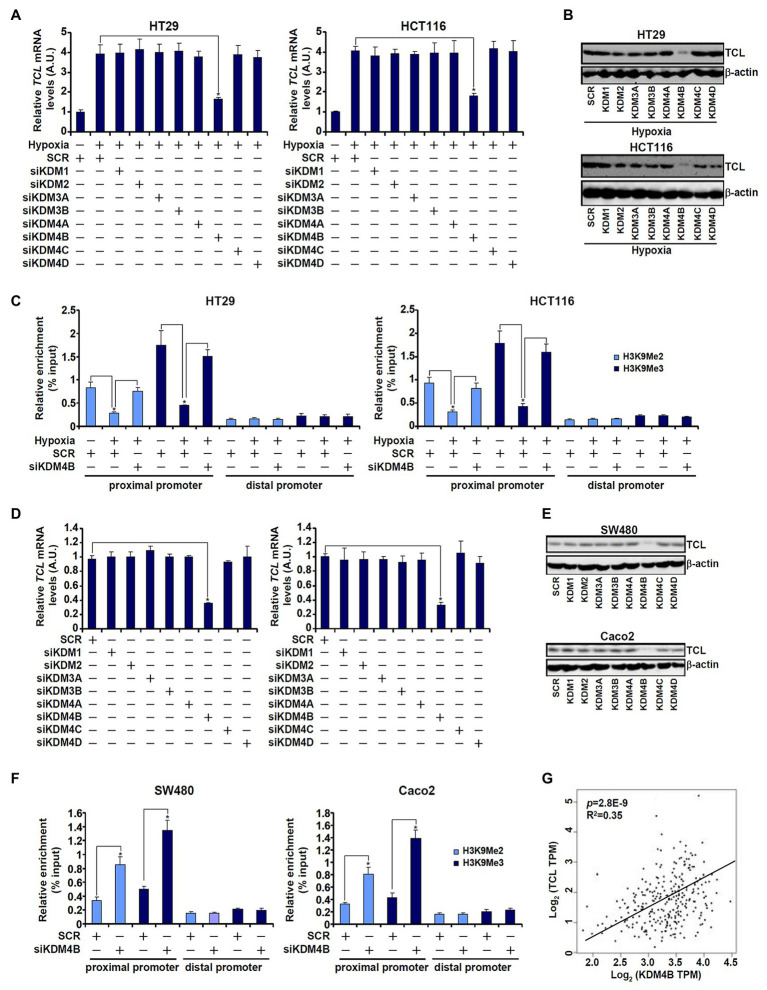
KDM4B regulates TCL transcription in CRC cells. **(A–C)** HT29 and HCT116 cells were transfected with indicated small interfering RNAs (siRNAs) followed by exposure to hypoxia. TCL expression levels were examined by qPCR and Western. ChIP assays were performed with anti-H3K9Me2 and anti-H3K9Me3. **(D–F)** SW480 and Caco2 cells were transfected with indicated siRNAs. TCL expression levels were examined by qPCR and Western. ChIP assays were performed with anti-H3K9Me2 and anti-H3K9Me3. **(G)** Expression data of KDM4B and TCL were extracted from TGCA to draw the scatter plot. Pearson correlation co-efficient was calculated.

Based on the observation that low H3K9 methylation levels correlated with high TCL expression in CRC cells, we hypothesized that a specific histone demethylase may be responsible for the regulation of differential TCL transcription. To verify this hypothesis, siRNAs targeting several individual KDMs were transfected into CRC cells. As shown in Figures 2A,B, out of the nine KDMs (Supplementary Figure S1 for knockdown efficiencies) targeted KDM4B knockdown markedly attenuated induction of TCL expression by hypoxia. Concordantly, KDM4B knockdown restored H3K9 methylation accumulation surrounding the TCL promoter (Figure 2C). Likewise, basal levels of TCL expression in SW480 and Caco2 cells, which were higher than those in HT29 and HCT116 cells under normoxic conditions, were downregulated only by KDM4B knockdown (Figures 2D,E). ChIP assay confirmed that both dimethylated and trimethylated H3K9 levels surrounding the TCL promoter were augmented in the absence of KDM4B (Figure 2F). To rule out the potential off-target effect of KDM4B siRNA, a second pair of KDM4B was employed (Supplementary Figure S2 for knockdown efficiency). KDM4B knockdown by siRNA#2 similarly down-regulated TCL expression (Supplementary Figure S3) and upregulated dimethyl/trimethyl H3K9 levels on the TCL promoter (Supplementary Figure S4). Of interest, KDM4B protein levels were higher in SW480 cells and Caco2 cells than in HT29 cells and HCT119 cells whereas hypoxia stimulation markedly augmented KDM4B expression in HT29 cells and in HCT116 cells (Supplementary Figure S5). More important, when expression data for both TCL and KDM4B in CRC patients were extracted from publicly available cancer database (TCGA), a strong positive correlation was identified (Figure 2G).

### ERG1 Recruits KDM4B to the TCL Promoter

Being an epigenetic factor, KDM4 inherently lacks the ability to recognize specific DNA sequences (Ptashne, 2007). Instead, it must rely on sequence-specific transcription factors to be recruited to the promoters. We (Chen et al., 2020b) and others (Yuan et al., 2011) have previously shown that ERG1 binds to the proximal TCL promoter to activate TCL transcription. We thus proposed that KDM4B might bind to the TCL promoter by interacting with ERG1. The following experiments were performed to test this proposal.

**Figure 3:**
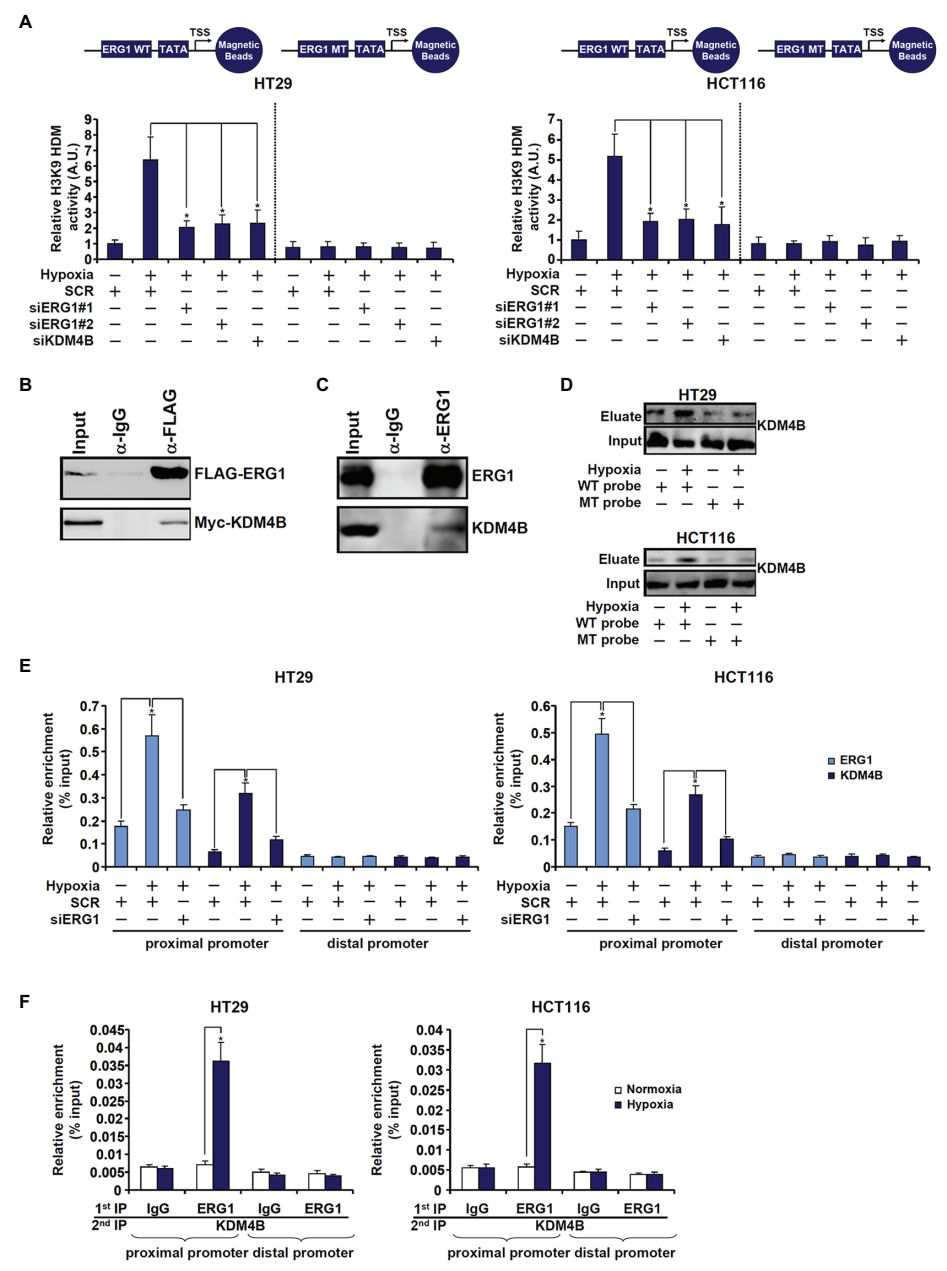
ETS-related gene 1 (ERG1) recruits KDM4B to the TCL promoter. **(A)** HT29 and HCT116 cells were transfected with indicated siRNAs followed by exposure to hypoxia. Nuclear proteins were extracted and incubated with the WT or the MT TCL probe. *In vitro* HDM assay was performed as described in the section “Materials and Methods.” **(B)** HEK293 cells were transfected with indicated expression constructs. Immunoprecipitation was performed with anti-FLAG or IgG. **(C)** HT29 cells were exposed to hypoxia for 24 h. Nuclear lysates were extracted and immunoprecipitation was performed with anti-ERG1 or IgG. **(D)** HT29 and HCT116 cells were transfected with indicated siRNAs followed by exposure to hypoxia. Nuclear lysates were extracted and DNA affinity pull-down assay was performed as described in the section “Materials and Methods.” **(E)** HT29 and HCT116 cells were transfected with indicated siRNAs followed by exposure to hypoxia. ChIP assays were performed with anti-KDM4B or anti-ERG1. **(F)** HT29 and HCT116 cells were treated with or without hypoxia for 24 h. Re-ChIP assay was performed with indicated antibodies.

First, DNA affinity pull-down experiments were performed to verify whether ERG1 might be associated with an H3K9 demethylase activity. As shown in Figure 3A, the DNA probe harboring wild type TCL promoter with an intact ERG1 site pulled-down strong H3K9 demethylase activity in hypoxia-exposed cells compared to normoxia-exposed cells. However, when the ERG1 site was mutated, the TLC promoter probe could no longer bring down any detectable H3K9 demethylase activity. Co-immunoprecipitation experiments confirmed that KDM4B was associated with ERG1 in HEK293 cells when both were over-expressed (Figure 3B) and in HT29 cells endogenously (Figure 3C). Further, DNA affinity pull-down experiments confirmed that hypoxia stimulation promoted the association of KDM4B with the wild type, but not the mutant, TCL promoter probe (Figure 3D). Importantly, ChIP assays showed that hypoxia enhanced the recruitment of both ERG1 and KDM4B to the proximal TCL promoter; ERG1 knockdown by siRNA dampened the abundance of both ERG1 and KDM4B detected on the proximal TCL promoter (Figure 3E). On the contrary, KDM4B depletion did not lead to significant changes in ERG1 binding on the TCL promoter (Supplementary Figure S6). Finally, Re-ChIP assay showed that an ERG1-KDM4B complex was detectable only in hypoxia-exposed CRC cells (Figure 3F).

### KDM4B Facilitates the Assembly of the Pre-initiation Complex on the TCL Promoter

**Figure 4:**
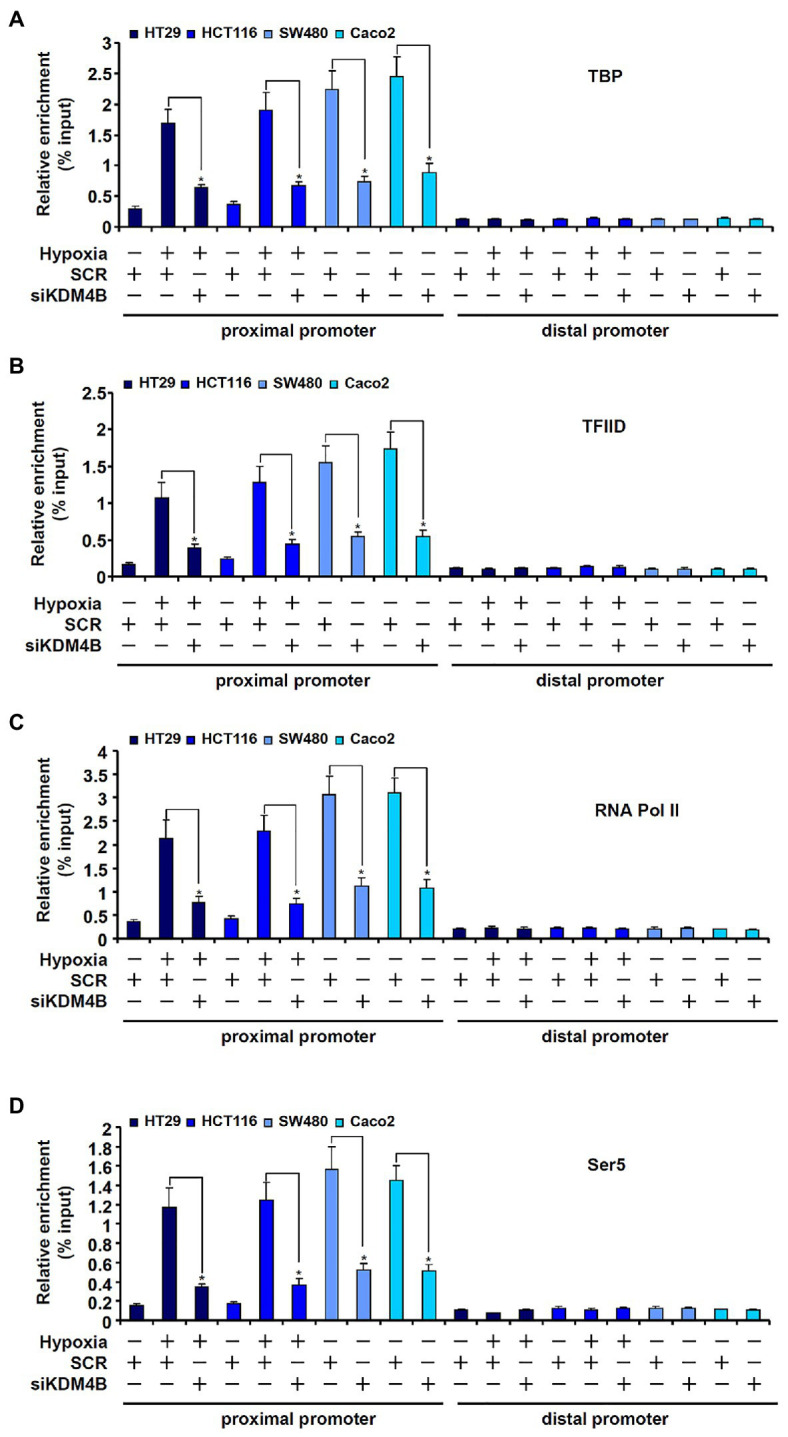
KDM4B facilitates the assembly of the pre-initiation complex (PIC) on the TCL promoter. **(A–D)** HT29 and HCT116 cells were transfected with indicated siRNAs followed by exposure to hypoxia. ChIP assays were performed with anti-TBP **(A)**, anti-TFIID **(B)**, anti-RNA Pol II **(C)**, and anti-Ser5 **(D)**.

Next, we examined the possible mechanism whereby KDM4B may regulate differential TCL transcription in CRC cells. The assembly of a PIC on gene promoters represents a key step in transcriptional regulation (Hahn, 2004). ChIP assays showed that basal (normoxic) levels of TATA binding protein (TBP; Figure 4A), transcription factor IID (TFIID; Figure 4B), and RNA Pol II (Figure 4C), key components of the PIC (Murakami et al., 2013), were much lower surrounding the TCL promoter in HT29 cells and HCT116 cells compared to SW480 cells and Caco2 cells. Moreover, hypoxia treatment of HT29 cells and HCT116 cells significantly increased the presence of TBP (Figure 4A), TFIID (Figure 4B), and RNA Pol II (Figure 4C) on the TCL promoter bringing their levels equivalent to those detected in SW480 cells and Caco2 cells. In addition, phosphorylation of serine 5 (Ser5) located on the C-terminal domain (CTD) of RNA Pol II, which serves as the rate-limiting step in transcription initiation (Harlen and Churchman, 2017), though lower in HT29 and HCT116 cells than in SW480 and Caco2 cells under normoxic conditions, was appreciably upregulated by hypoxia stimulation (Figure 4D). KDM4B knockdown, however, largely blocked the accumulation of the PIC factors on the TCL promoter (Figures 4A–D). Together, these data suggest that KDM4 may regulate TCL transcription by facilitating the assembly of the PIC on the TCL promoter.

### KDM4B Regulates CRC Cell Migration/Invasion and Is Associated With Poor Prognosis in CRC Patients

**Figure 5:**
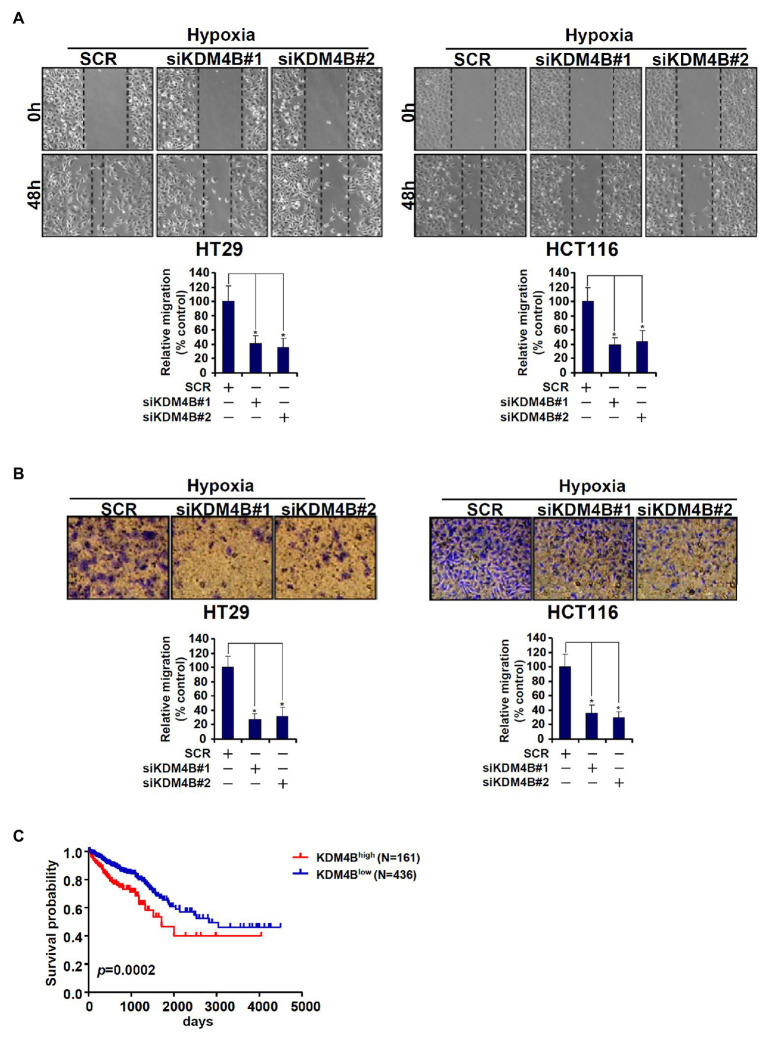
KDM4B regulates CRC cell migration/invasion and is associated with poor prognosis in CRC patients. **(A,B)** HT29 and HCT116 cells were transfected with indicated siRNAs followed by exposure to hypoxia. Wound healing assay and trans-well assay were performed and quantified as described in the section “Materials and Methods.” **(C)** Kaplan-Meier plot of survival in CRC patients with high and low KDM4B expression.

Because we have previously shown that TCL plays an important role in promoting CRC cell migration and invasion, we determined the functional relevance of KDM4B in this regard. As shown in Figure 5A, knockdown of KDM4B by two independent pairs of siRNAs comparably and significantly attenuated the migration of HT29 cells and HCT116 cells as measured by wound-healing assay. Boyden chamber trans-well assay showed that KDM4B depletion also weakened the invasion of HT29 and HCT116 cells (Figure 5B). Finally, when the Kaplan-Meier analysis was performed to correlate KDM4B expression with CRC patient survival it was discovered that high KDM4B levels in CRC patients were associated with a significantly poorer prognosis than low KDM4B levels (Figure 5C).

## Discussion

KDM4B is a member of the KDM4 lysine demethylase family with specificity toward di- and tri-methylated H3K9 (Berry and Janknecht, 2013). KDM4B has been shown to contribute to cancer development and progression by regulating the transcription of a myriad of cancer-related genes (Wilson and Krieg, 2019). In the present report, we have identified KDM4B as a novel transcriptional activator of the *TCL* gene, which encodes a pro-metastatic small GTPase, in CRC cells. Although our analysis indicates that elevated KDM4B expression in CRC patients predicts poor survival (Figure 5C), we suspect that KDM4B exerts its pro-oncogenic effects solely *via* activating TCL transcription. Li et al. (2020a), for instance, have recently reported that KDM4B regulates glucose transporter 1 (GLUT1) expression in CRC cells to drive glycolysis-fueled proliferation. Chen et al. (2014) and Deng et al. (2018) have independently shown that KDM4B is essential for DNA damage repair and resistance to radiotherapy in CRC cells by regulating STAT3 transcription. Additionally, KDM4B could act as a co-factor for β-catenin, a prototypical colorectal oncogene, to activate the transcription of genes involved in cell proliferation and migration (Berry et al., 2014; Sun et al., 2020). Therefore, it is likely that KDM4B contributes to CRC malignancies by regulating a panel of target genes including TCL. A transcriptomic study by Wilson et al. (2017) conducted in ovarian cancer cells shows that KDM4B selectively regulates the transcription of genes involved in cell movement under hypoxic conditions; whether a similar scenario takes place in CRC cells remains to be determined. Future studies should exploit transcriptomic techniques, RNA-seq, and ChIP-seq, for example, to uncover the full spectrum of KDM4B-dependent transcriptional events in CRC cells.

We show here that KDM4B activates TCL transcription by interacting with ERG1. There are multiple reports that point to a functional interplay between ERG1 and the epigenetic machinery. Yang et al. (2002) were among of the first to suggest that ERG1 regulates transcription in cancer cells in part by interacting with an H3-specific methyltransferase ESET. A re-occurring theme of ERG1-dependent transcription in prostate cancers is its interaction with the H3K27 methyltransferase complex PRC although it is not clear whether similar mechanisms are operative in other cancers (Sreenath et al., 2011). Kim et al. (2016b) have shown that ERG1 can potentially activate the transcription of proto-oncogene YAP by cooperating with KDM4A. More recently, Kalna et al. (2019) demonstrate that ERG1 is essential for the maintenance of certain lineage-specific enhancers/super-enhancers presumably by recruiting an H3K27 acetyltransferase. Although ERG1 was initially described in human CRC cells as a homolog for the ETS proto-oncogene (Reddy et al., 1987), few studies exist that provide mechanistic insights into the regulatory role of ERG1 in CRC development and progression. Our data certainly serve as renewed incentive to further investigate how ERG1 harnesses the epigenetic machinery, KDM4B included, to program malignant transformation of CRC cells.

We propose that KDM4B activates TCL transcription by facilitating the assembly of the RNA polymerase II PIC. Two issues warrant further attention. First, although it is generally agreed that the recruitment of RNA polymerase II PIC is only possible on a compatible chromatin microenvironment defined by specific histone and DNA modifications (Gates et al., 2017), it is unlikely that this process is solely determined by KDM4B-mediated H3K9 demethylation. Rather, a more plausible model would be that a multi-protein complex including KDM4B coordinates, *via* extensive crosstalk, the modulation of chromatin structure to facilitate PIC assembly. Whetstine and colleagues have recently reported KDM4B, in cooperation with the H3K4 methyltransferase MLL and the H3K4 demethylase KDM5B, controls site-specific DNA amplification (Mishra et al., 2018). We have shown that there is crosstalk between KDM4B and the chromatin remodeling protein BRG1 in mediating the Wnt/β-catenin signaling pathway (Li et al., 2019a). Whether a similar mechanism is associated with TCL trans-activation in CRC cells remains to be elucidated. Second, KDM4B could potentially contribute to TCL transcription and drive CRC malignancy through demethylating non-histone substrates. Recently, Ponnaluri et al. (2009) have reported that purified KDM4B could use several pro-oncogenic proteins, such as G9a and CDYL1, as substrates although the functional relevance of this finding is unclear. Studies along this line to provide further mechanistic insight into the way KDM4B regulates transcription and disease pathogenesis are warranted.

In summary, our data here unveil a novel epigenetic mechanism underlying malignant transformation of CRC cells. There are a few limitations regarding our study. First, TCL expression was not compared between normal intestinal epithelial cells and various CRC cells. Therefore, it remains unknown whether TCL could contribute to the malignant transformation of intestinal epithelial cells. Second, although we show that there appears to be an inverse correlation between KDM4B expression and survival rate of CRC patients, it cannot be concluded with certainty that KDM4B levels can be used as an independent marker for CRC prognosis due to the small sample size. Further analysis to include more patient data is needed to solidify the prognostic value of KDM4B. Small-molecule KDM4B inhibitors have been designed and demonstrated to be effective in cell culture (Chen et al., 2017; Lin et al., 2018). Our data certainly provide renewed rationale for exploiting KDM4B as a druggable target in CRC intervention.

## Data Availability Statement

The original contributions presented in the study are included in the article/Supplementary Material; further inquiries can be directed to the corresponding author.

## Author Contributions

YF, BC, YZ, and JC designed and performed the experiments, and collected and analyzed the data. JC and YF provided the funding. YX wrote the manuscript. All authors contributed to the article and approved the submitted version.

### Conflict of Interest

The authors declare that the research was conducted in the absence of any commercial or financial relationships that could be construed as a potential conflict of interest.
