# Supplementary material for: Activation of TC10-Like Transcription by Lysine Demethylase KDM4B in Colorectal Cancer Cells
Source: Front Cell Dev Biol. 2021 Jun 23;9:617549. doi: 10.3389/fcell.2021.617549 (PMC8260841; doi:10.3389/fcell.2021.617549)
Supplement: Supplementary file 1 [file Data_Sheet_1.PDF]

Chen BY et al: Activation of TCL transcription by lysine demethylase KDM4B in colorectal cancer cells

Online supplementary material

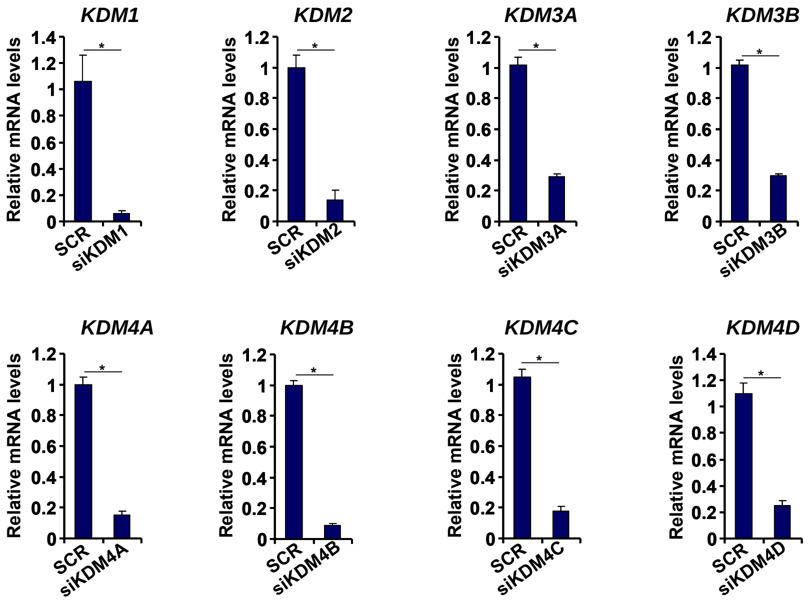

Figure S1: Knockdown efficiencies of different KDMs in HT29 cells were verified by qPCR.

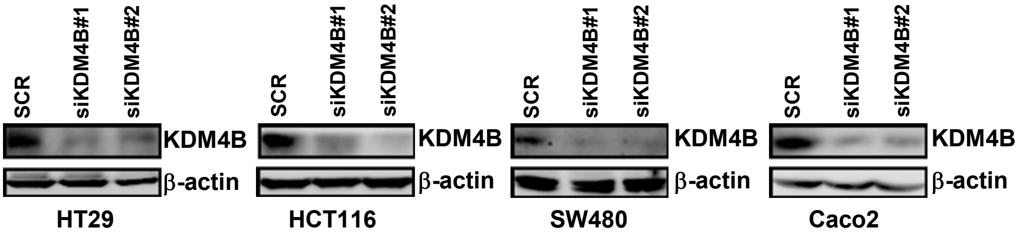

Fig.S2: Knockdown efficiencies of different KDMs in HT29 cells were verified by qPCR.

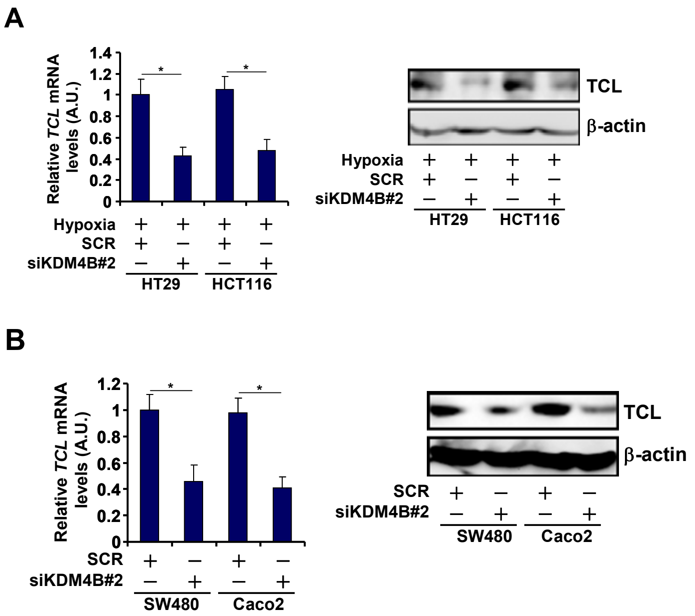

Fig.S3: (**A**) HT29 and HCT116 were transfected with indicated siRNAs followed exposure to hypoxia. TCL expression levels were examined by qPCR and Western. (**B**) SW480 and Caco2 cells were transfected with indicated siRNAs. TCL expression levels were examined by qPCR and Western.

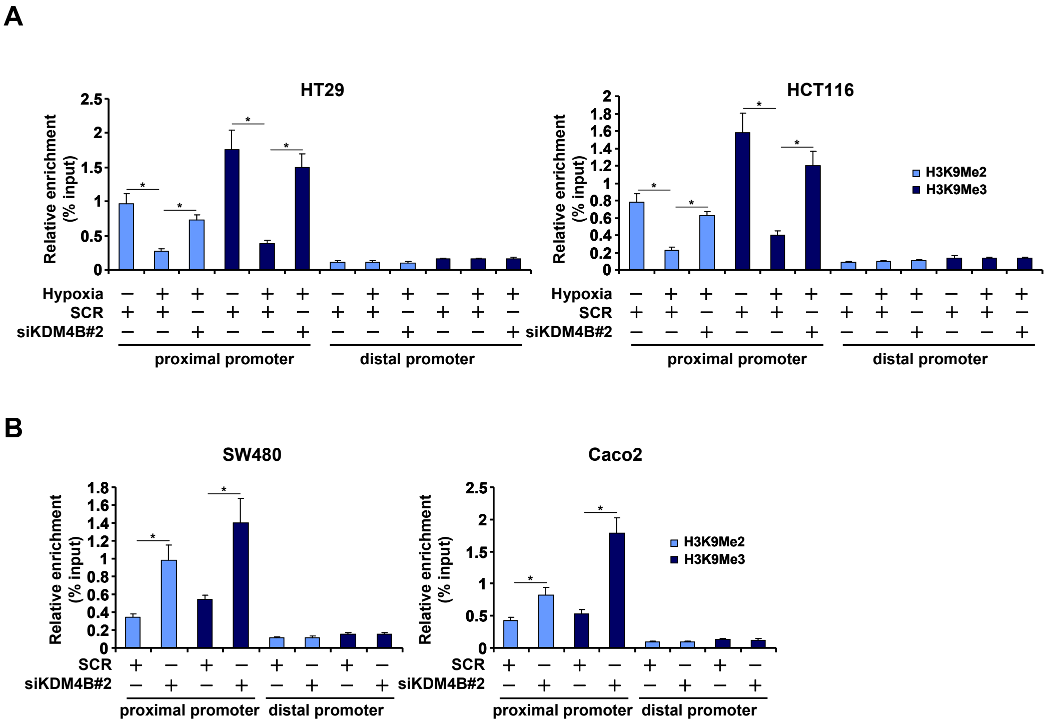

Fig.S4: (**A**) HT29 and HCT116 were transfected with indicated siRNAs followed exposure to hypoxia. ChIP assays were performed with anti-dimethyl H3K9 and anti-trimethyl H3K9. (**B**) SW480 and Caco2 cells were transfected with indicated siRNAs. ChIP assays were performed with anti-dimethyl H3K9 and anti-trimethyl H3K9.

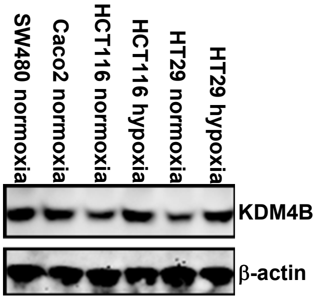

Fig.S5: CRC cells were exposed to hypoxia or normoxia for 24h. KDM4B expression was examined by Western.

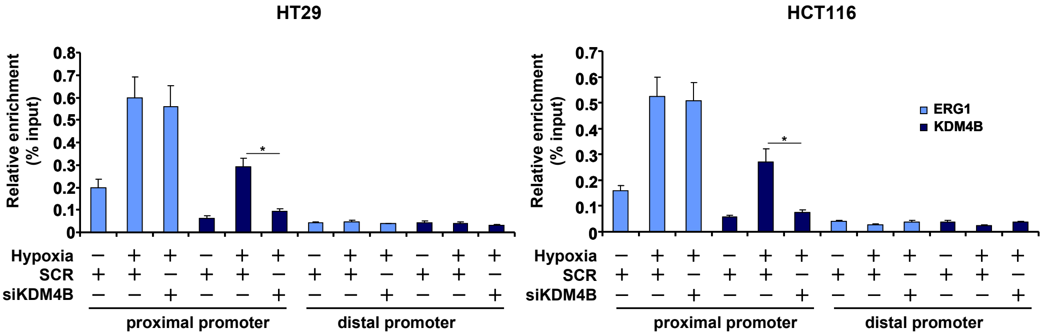

Fig.S6: HT29 and HCT116 cells were transfected with indicated siRNAs followed by exposure to hypoxia. ChIP assays were performed with anti-KDM4B or anti-ERG1.
